# Differential effects of soluble and aggregating polyQ proteins on cytotoxicity and type-1 myosin-dependent endocytosis in yeast

**DOI:** 10.1038/s41598-017-11102-6

**Published:** 2017-09-12

**Authors:** Lisa L. Berglund, Xinxin Hao, Beidong Liu, Julie Grantham, Thomas Nyström

**Affiliations:** 1 Institute of Biomedicine – Department of Microbiology & Immunology, Sahlgrenska Academy, University of Gothenburg, Medicinaregatan 7A, 405 30 Göteborg, Sweden; 2 Department of Chemistry and Molecular Biology, University of Gothenburg, Medicinaregatan 9C, 405 30 Göteborg, Sweden

## Abstract

Huntington’s disease develops when the polyglutamine (polyQ) repeat in the Huntingtin (Htt) protein is expanded to over 35 glutamines rendering it aggregation-prone. Here, using Htt exon-1 as a polyQ model protein in a genome-wide screen in yeast, we show that the normal and soluble Htt exon-1 is toxic in cells with defects in type-1 myosin-dependent endocytosis. The toxicity of Htt is linked to physical interactions with type-1 myosins, which occur via the Htt proline-rich region, leading to a reduction in actin patch polarization and clathrin-dependent endocytosis. An expansion of the polyQ stretch from 25 to 103 glutamines, which causes Htt aggregation, alleviated Htt toxicity in cells lacking Myo5 or other components involved in early endocytosis. The data suggest that the proline-rich stretch of Htt interacts with type-1 myosin/clathrin-dependent processes and demonstrate that a reduction in the activity of such processes may result in a positive selection for polyQ expansions.

## Introduction

Huntington’s disease (HD) is one of several neurological diseases caused by polyQ expansions in specific proteins. In the case of HD, CAG trinucleotide repeats causing the polyQ expansion occurs in exon-1 in the Huntingtin (HTT) gene^1^. Expression of polyQ-extended exon-1 leads to the formation of intracellular aggregates/inclusion bodies (IBs) in mice and the animals develop symptoms with similar characteristics to those of patients with HD^2, 3^. Furthermore, IBs detected in brains of deceased HD patients contain fragments of Htt similar to exon-1. These fragments of Htt are generated through proteolytic cleavage of the full-length protein^4^. In yeast, the aggregation pattern of Htt exon-1 mimics that of mammalian cells; the protein with a glutamine stretch of 25 repeats is soluble in the cytoplasm, while repeat lengths of 47 or more lead to protein aggregation and the formation of typical IBs^5^. Initially, such IBs of exon-1 were suggested to be the cause of HD, but recent studies have suggested that smaller exon-1 oligomers/aggregates are the toxic conformers^6–8^. In line with this notion, chaperones, peptides, and prion-like proteins that either prevent/reduce oligomer production^5, 9–12^ or convert small aggregates/oligomers into IBs^13, 14^ can suppress the toxicity of the exon-1 fragment^6, 15^. Htt toxicity is also dependent on sequences flanking the polyQ repeat^12, 16^. For example, the removal of the proline-rich region directly C-terminal of the expanded polyQ stretch (Htt103Q) results in the formation of multiple amorphous-like aggregates rather than one or two large inclusions^12^. Interestingly, these amorphous aggregates of Htt103Q are severely toxic in yeast cells, whereas the inclusions of the native, proline-rich region-containing Htt103QP, are not^12^. However, it is not clear how this involvement of the proline-rich region in relieving Htt toxicity is pertinent to HD as the proline-rich region is supposedly always present in exon-1.

In this study, using Htt exon-1 as a model polyQ protein and yeast as a model organism, we performed a genome-wide screen for polyQ toxicity and found that soluble exon-1 interacts both genetically and physically, in a proline-rich region-dependent manner, with type-1 myosin motor proteins (Myo3/Myo5). Our analysis further revealed that soluble Htt exon-1 is highly toxic in cells with a reduced activity in early, actin- and type-1 myosin-dependent, endocytosis and that aggregation of Htts with polyQ expansions mitigates such toxicity. The results imply that in some genetic landscapes, the expansion of polyQ to aggregation-prone, disease-related, Htts might be under positive selection.

## Results

### Htt exon-1 is toxic in yeast cells defective in actin nucleation

**Figure 1:**
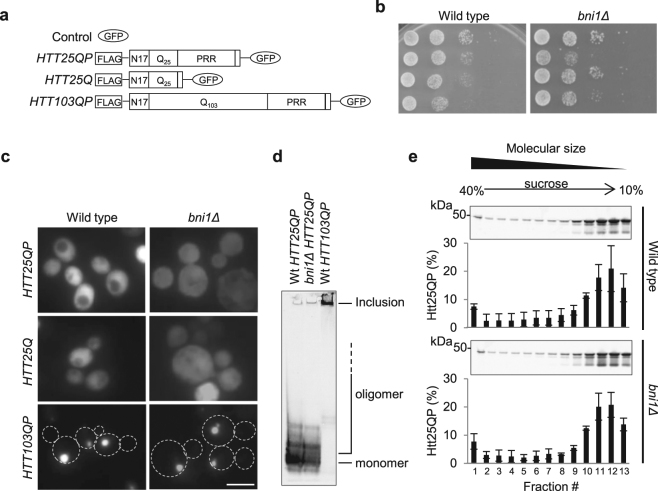
The yeast formin Bni1 is important for Htt25QP detoxification. (**a**) Schematic representation of the constructs used with and without the proline-rich region (PRR). (**b**) 10x serial dilutions of wild type and *bni1Δ* yeast cells expressing indicated constructs. Representative images from ≥ two individual experiments are shown. (**c**) Fluorescence micrographs of exponentially growing wild type (left) and *bni1Δ* (right) yeast cells expressing indicated *HTT* constructs. Scale bar = 5 µm. (**d**) Native PAGE of protein extracts from wild type and *bni1Δ* cells expressing indicated *HTT* constructs. Representative picture from three independent experiments is shown. (**e**) Sucrose density gradient fractionation of protein extracts from wild type and *bni1Δ* cells expressing *HTT25QP* followed by Western blot analysis. The percentage of Htt25QP protein present in each fraction is quantified in graphs below each Western blot. Values are averages from three independent experiments with error bars corresponding to the standard deviation. (Full-length blots are shown in Fig. S12).

The *HTT* constructs used in this study for ectopic expression in yeast are presented in Fig. 1a. In our toxicity studies of expanded polyQ Htts in different yeast mutants, we noticed that the typically non-toxic and soluble, wild type, Htt25QP was in fact more toxic than Htt103QP in cells lacking the formin Bni1 (Fig. 1b). Fluorescence microscopy did not reveal any Htt25QP foci (aggregates) and the expression pattern of the *GFP*-tagged exon-1 constructs in the *bni1Δ* strain was similar to that seen in wild type cells (Fig. 1c), indicating that the toxicity of Htt25QP was not due to aggregate formation or elevated expression. To determine if the Htt25QP protein in a *bni1Δ* strain formed toxic oligomers too small to be detected by normal fluorescence microscopy, we performed native gel electrophoresis and sucrose density gradient ultracentrifugation of protein extracts from a wild type and a *bni1Δ* strain, each expressing *HTT25QP* and found that there was no discernable difference in Htt25QP oligomer composition between the two strains (Fig. 1d and e). This suggests that the toxicity of Htt25QP in *bni1Δ* cells is unlinked to protein aggregation/oligomer formation. As expected, the Htt103QP protein formed almost exclusively large, SDS-insoluble, inclusions (Fig. 1d).

### Reduced function in actin-dependent endocytosis renders Htt25QP, but not Htt25Q, toxic

**Figure 2:**
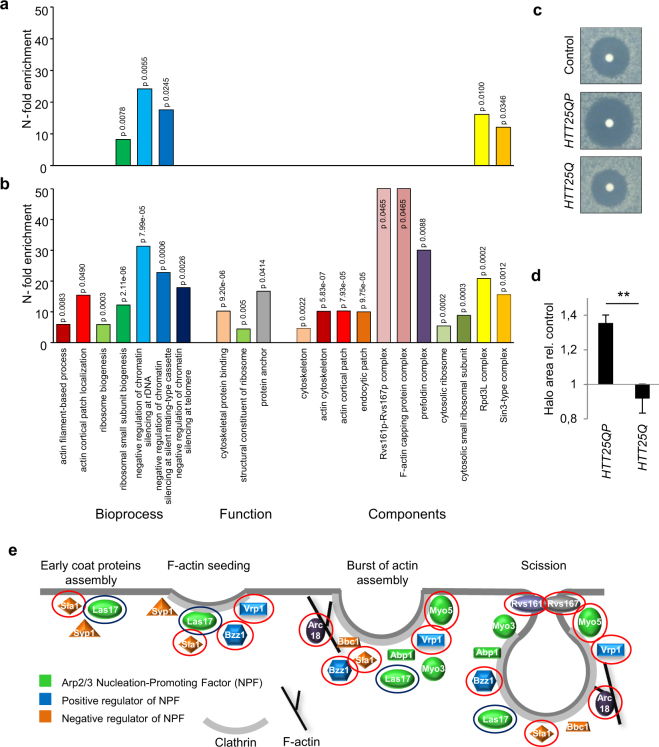
A functional actin cytoskeleton is important for mitigating Htt25QP toxicity. Functional enrichment analysis of (**a)** *HTT25Q* and (**b**) *HTT25QP* genetic interactions. (**c**) Latrunculin A sensitivity test of wild type cells expressing indicated constructs. (**d**) Quantification of halo area of no growth from three individual experiments in **c**. Error bars correspond to the standard deviation, *P* = 0.0014. (**e**) Schematic drawing of proteins involved in actin patch formation and early endocytosis that are represented in the SGA screening library. Proteins with a red circle are encoded by genes that displayed a negative genetic interaction with *HTT25QP* but not with *HTT25Q*. The blue circle indicates a manually selected *las17* ts allele from the essential library showing a negative genetic interaction with *HTT25QP* but not with *HTT25Q*.

To determine if the deletion of other genes rendered soluble Htt25QP toxic, we performed a synthetic genetic array (SGA) screen to uncover processes affected by Htt. *HTT25QP* and *HTT25Q* were crossed into a yeast deletion library and genes showing negative genetic interactions with *HTT25QP* and *HTT25Q* are presented in supplementary Table S1. Gene ontology and functional enrichment analysis demonstrated that while *HTT25Q* displayed negative genetic interactions with genes involved in ribosome biogenesis and chromatin regulation (Fig. 2a), *HTT25QP* displayed many additional interactions, including negative genetic interactions with genes encoding actin cytoskeleton components (Fig. 2b). To verify the link between Htt25QP and the actin cytoskeleton, we tested the sensitivity to the actin monomer-sequestering drug Latrunculin A, which leads to depolymerization of F-actin structures (rings, cables and patches) and found that expression of *HTT25QP* resulted in an increased Latrunculin A sensitivity compared to the control and *HTT25Q* (Fig. 2c and d). Expression of *HTT25QP* appears to affect the actin cytoskeleton rather than the microtubule network as the microtubule depolymerizing drug benomyl, did not result in increased sensitivity to Htt25QP (Fig. S1).

### Htt25QP impedes endocytosis in yeast cells deficient in type-1 myosin motor activity

Genes involved in the formation of cortical actin patches and clathrin-mediated endocytosis, including the type-1 myosin *MYO5*, were highly enriched among the genetic interactors of *HTT25QP* (Fig. 2e).

**Figure 3:**
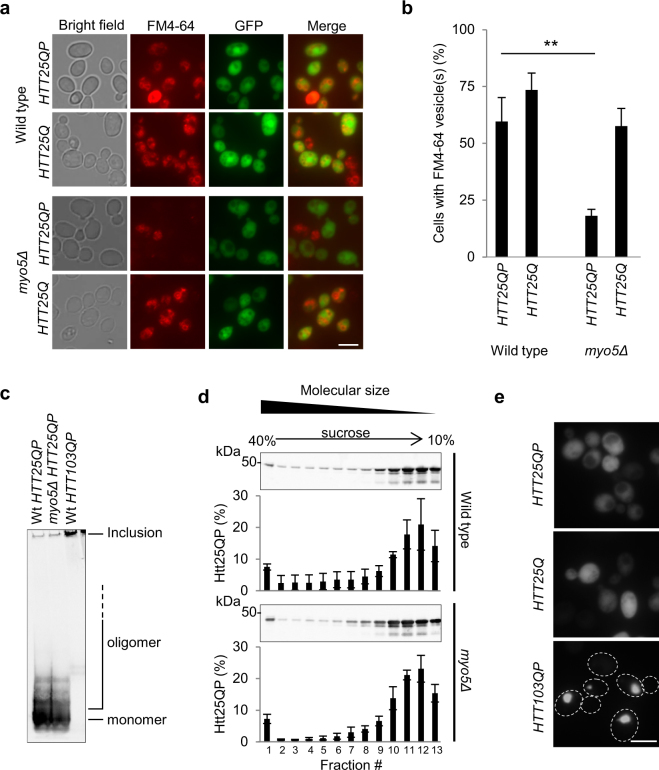
Soluble Htt25QP blocks endocytosis in cells lacking Myo5. (**a**) Images of wild type and *myo5Δ* cells expressing *HTT25QP* and *HTT25Q* and their capacity of endocytic uptake. Scale bar = 5 µm. (**b**) Quantification of **a**. Percentage of cells with detectable Htt levels capable of internalizing FM4-64 via the endocytic pathway. Average values from three individual experiments, with more than 150 cells per experiment, are shown. Error bars corresponds to the standard deviation, *P* = 0.00284. (**c**) Native PAGE of protein extracts from wild type and *myo5Δ* cells expressing *HTT* constructs. Representative picture from three independent experiments is shown. (**d**) Sucrose density gradient fractionation of protein extracts from wild type and *myo5Δ* cells expressing *HTT25QP*. The percentage of Htt25QP protein present in each fraction is quantified in graphs below each Western blot. Values are average from three independent experiments with error bars corresponding to the standard deviation. (Full-length blots are shown in Fig. S12) (**e**) Fluorescence micrographs of exponentially growing *myo5Δ* yeast cells expressing indicated *HTT* constructs. Scale bar = 5 µm.

Myo5 is one of the two type-1 myosin motor proteins (Myo5 and Myo3) in yeast and is a central component in early clathrin-dependent endocytosis and actin patch formation. Therefore, we tested the effect of Htt25QP and Htt25Q on endocytosis in cells lacking *MYO5*, demonstrating that without Myo5, Htt25QP, but not Htt25Q, dramatically reduces endocytic uptake of FM4-64 (Fig. 3a and b) after the dye had being incorporated in the plasma membrane (Fig. S2). The *myo5∆* mutation on its own did not affect cellular growth rate (Fig. S10) or the rate of endocytosis (see below). The data indicates that the proline-rich region is required for Htt to affect actin-dependent endocytosis (Fig. 3a and b). In the *myo5Δ* mutant cells (as was the case for *bni1Δ* cells), Htt25QP toxicity was not linked to increased aggregate (Fig. 3a) or oligomer (Fig. 3c and d) formation. Htt25QP did not form aggregates in the *myo5∆* mutant (Fig. 3e), similar to the *bni1∆* mutant (Fig. 1c).

### Type-I myosins bind specifically to Htt25QP

**Figure 4:**
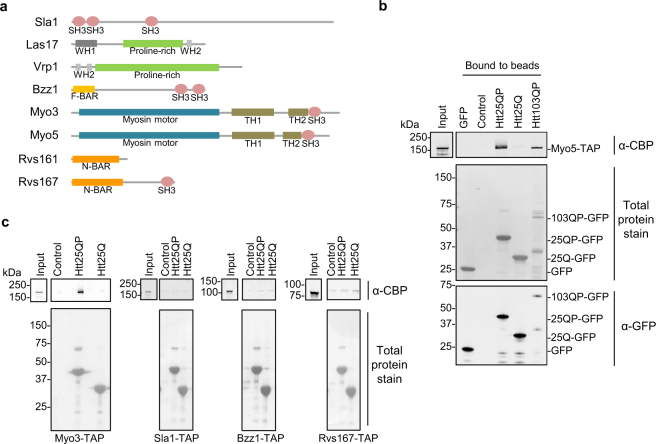
Type-1 myosins interact with Htt25QP. (**a)** Schematic picture showing protein domains of endocytic factors important for detoxifying Htt25QP containing either proline-rich regions themselves, and/or proline-rich region-binding domains. (*MYO3* did not show genetic interactions with *HTT25QP*, but is shown due to its redundancy with *MYO5* and data presented below.) Illustrations are made using Pfam and Prosite. The proline-rich sequences are manually created. (**b**) and (**c**) *In vitro* binding assay between immobilized GFP proteins on GFP-Trap agarose beads and indicated SH3 domain-containing proteins. Blots are stained with PonceauS for visualization of total proteins and probed for TAP-tagged proteins using an anti-CBP antibody and GFP tagged proteins using an anti-GFP antibody. Representative pictures from two (Sla1, Bzz1, and Rvs167) or three (Myo5 and Myo3) independent experiments are shown (the binding of Myo5 to Htt103QP was analyzed two times). (Full-length blots are shown in Fig. S12).

The fact that the Htt toxicity in the cytoskeletal mutants identified was seen only for Htt25QP and not Htt25Q, suggests that the proline-rich region is important for interactions, genetic and/or physical, with proteins involved in actin/clathrin-dependent endocytosis. We therefore hypothesized that Htt25QP may sequester proteins required for actin-dependent endocytosis and when removing one component of this process, by deleting the corresponding gene, Htt25QP may become more toxic since more Htt25QP would be available for interaction with the residual components of actin/clathrin-dependent endocytosis. It is known that proline-rich regions can bind SH3 domains^17, 18^. Interestingly, proteins encoded by several of the negative genetic interactors of *HTT25QP* identified in our SGA screen contain one or several SH3 domains (Fig. 4a).

We tested these proteins in an *in vitro* binding assay and found that Myo5 bound to Htt25QP, whereas no specific binding to Htt25Q could be detected (Fig. 4b), suggesting that the poline-rich region is required for myosin binding. Whether the proline-rich region is sufficient for binding is not presently known, however. Other SH3 domain-containing proteins than Myo5 identified in our SGA screen, Sla1, Bzz1, and Rvs167, did not show a specific binding to Htt25QP. Similar to Myo5, the other type-1 myosin, Myo3, was also found to bind to Htt25QP but not to Htt25Q (Fig. 4c). This suggests that the interaction between type-1 myosins and the proline-rich region of Htt25QP involves either additional regions to the SH3 domain or other type-1 myosin specific regions. Cells lacking Myo3, however, did not show a negative genetic interaction with *HTT25QP* in the SGA screen and did not exhibit sensitivity towards Htt25QP when tested manually (Fig. S3). This can either be explained by Myo5 being more abundant than Myo3^19–22^ or that Myo5 possesses some functions absent in Myo3 (see below)^23^. A double *myo5* and *myo3* deletion has been reported previously to cause severe defects in both growth and endocytosis^24^. We next tested if the polyQ extended Htt (Htt103QP) in its soluble form was still capable of binding Myo5 and found this to be the case (Fig. 4b).

### Htt25QP titrates type-1 myosins leading to defects in actin polarity and endocytosis

**Figure 5:**
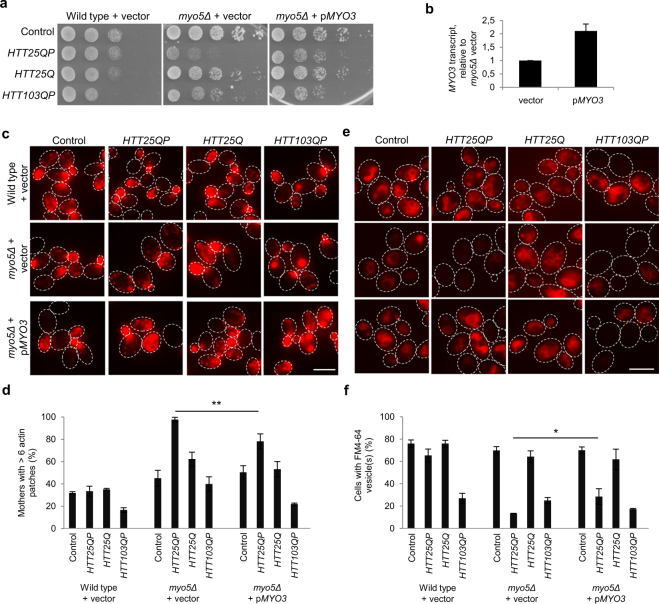
Htt25QP limits type-1 myosin activity. The depolarized state and reduced endocytosis of *myo5Δ* Htt25QP-producing cells can be partially suppressed by overexpressing the other type-1 myosin *MYO3*. (**a**) 5x serial dilutions of wild type and *myo5Δ* cells expressing indicated constructs. Representative images from three individual experiments are shown. (**b**) *MYO3* transcript levels normalized to *myo5Δ* + vector in cells harboring *HTT25QP* plasmid. Values are average from two independent experiments with error bars corresponding to the standard deviation. (**c**) Fluorescence micrographs of wild type and *myo5Δ* cells expressing different constructs used in this study and stained for F-actin structures. Maximum projection is shown. Scale bar = 5 µm. (**d**) Quantification of **c**. Graph shows percentage mother cells having more than six actin patches. Cells not expressing *HTT* fragments at detectable levels are excluded from the analysis. A total of ten Z-stack images were analyzed per field of view. Average values from three individual experiments using two different clones with more than 150 cells per experiment are shown. Error bars correspond to the standard deviation, *P* = 0.0087. (**e**) Fluorescence micrographs showing the cells’ capability of endocytic uptake. Scale bar = 5 µm. (**f**) Quantification of **e**. Graph shows percentage cells with endocytic vesicle(s). Cells not expressing *HTT* fragments at detectable levels are excluded. Average values from three individual experiments using two different clones with more than 150 cells per experiment are shown. Error bars correspond to the standard deviation, *P* = 0.020.

As both Myo5 and Myo3 are important for the polarization of the actin cytoskeleton and endocytosis, these proteins were analyzed further with regard to their involvement in Htt25QP toxicity. We tested if the expression of *HTT25QP* is affecting the localization of Myo5 within the cell. During CME, type-1 myosins are recruited to endocytic sites (patches) at the plasma membrane to positively regulate actin polymerization^24, 25^. When comparing wild type yeast cells with an mRuby-tagged Myo5, either expressing *HTT25QP* or *HTT25Q*, we found that the presence of Htt25QP led to a reduction in Myo5 patches at the plasma membrane compared to Htt25Q (Fig. S4). Further, we found that overexpression of *MYO3* partly suppressed the toxicity of Htt25QP in *myo5Δ* cells, suggesting that soluble Htt25QP is sequestering type-1 myosins, preventing them from performing their normal functions in the cell (Figs 5a,b and S5). In addition, we found that the reduced polarization (Figs 5c and d and S6) and endocytic uptake (Figs 5e and f and S7) displayed by *myo5Δ* cells expressing *HTT25QP* could be partly suppressed by overexpression of *MYO3* (Fig. 5c,d,e,f and Figs S6 and S7). As described previously^26^, Htt103QP caused a collapse in the actin cytoskeleton and reduced endocytic uptake on its own but endocytosis was not further reduced by deleting *MYO5* and could not be suppressed by overexpressing *MYO3* (Fig. 5e and f). Thus, we conclude that Htt103QP is causing problems in endocytosis by a mechanism distinct from that of Htt25QP. Co-staining for Htt25QP-GFP or Htt103QP-GFP clearly showed that it was the cells with high expression of either *HTT25QP* or *HTT103QP* that had severe problems in endocytosis (Fig. S7), after FM4-64 had been incorporated into the plasma membrane (Fig. S8).

### The pattern of Htt polyQ length-dependent toxicity is altered in cytoskeleton/endocytosis mutants

**Figure 6:**
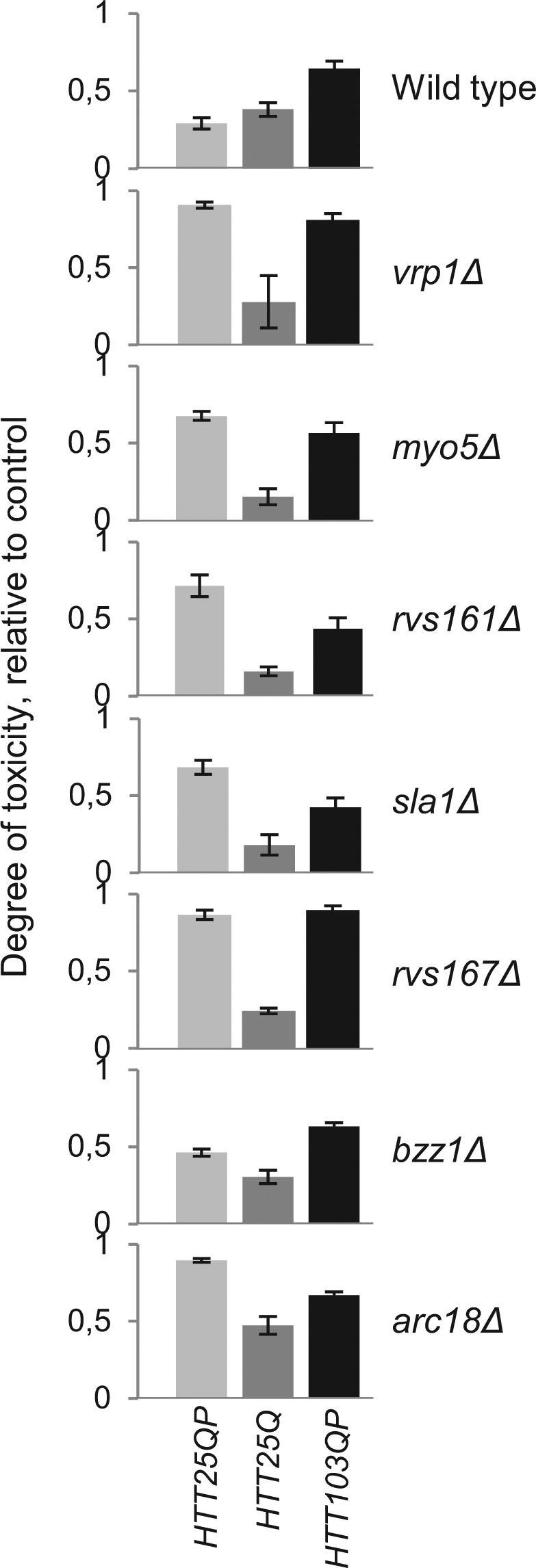
Differential toxicity of Htt25QP and Htt103QP in cells with reduced activities in actin dependent endocytosis. Exponentially growing cultures were spotted onto appropriate solid media containing galactose for induction of Htt proteins and allowed to grow. Cell growth of each spot was quantified and values relative to the control within each strain are shown as the degree of polyQ toxicity. Values are from three individual experiments with error bars corresponding to the standard error of the mean.

Similar to *bni1Δ* cells, the cytoskeleton/endocytosis mutants identified in the screen displayed an altered pattern of sensitivity to the different *HTT* exon-1 constructs, in comparison to wild type cells, as revealed by toxicity tests on both plates (Figs 6 and S9) and in liquid (Fig. S10). Apart from the fact that the proline-rich region was required for toxicity in all mutants tested, the Htt25QP protein displayed a higher degree of toxicity compared to the polyQ expanded variant Htt103QP in many of the mutants (Figs 6 and S9). There were no mutant strains among the ones tested that showed a significantly higher level of the Htt proteins compared to the wild type strain (Fig. S11), demonstrating that the growth reduction observed was not a trivial result of elevated expression.

### Htt polyQ expansions may be selected in cytoskeleton/endocytosis mutants

**Figure 7:**
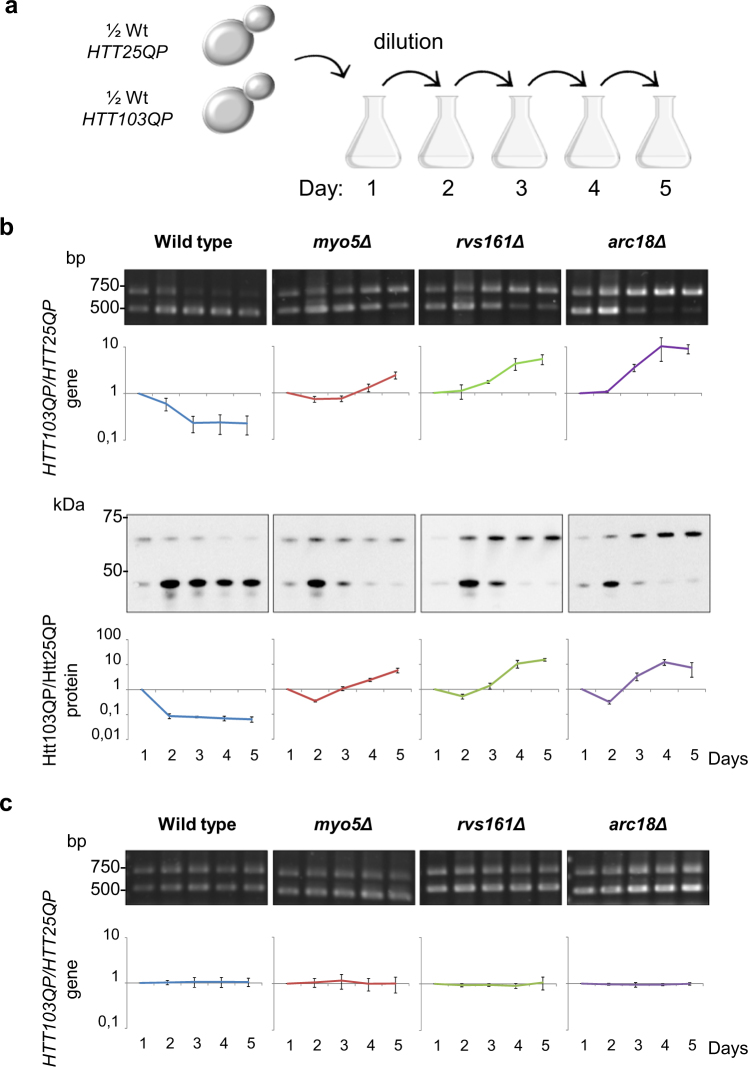
Polyglutamine expansion alleviates Htt25QP toxicity in cells with mutations in clathrin-mediated endocytosis. (**a**) Experimental outline for competition assay. Equal cell numbers from *HTT25QP* and *HTT103QP* isogenic strains were mixed and grown for five days with sampling and dilution of the culture once every 24 hours. (**b**) Mixed cultures were grown with galactose as carbon source to allow for induction of Htt proteins. Agarose gels with PCR products quantified as the *HTT103QP*/*HTT25QP* gene ratio (top) and Western blots with total protein extracts quantified as the Htt103QP/Htt25QP protein ratio (bottom). Values are average from three individual experiments with error bars corresponding to the standard deviation. (**c**) Mixed cultures were grown with raffinose as carbon source and used as uninduced controls. Agarose gels with PCR products quantified as the *HTT103QP*/*HTT25QP* gene ratio. Values are average from three individual experiments with error bars corresponding to the standard deviation. (Full-length gels and blots are shown in Fig. S13).

The data presented raise the question of whether there may be an evolutionary pressure for polyQ expansion in some cells and tissues depending on the activity and/or abundance of their endocytic machinery. To address if the aggregating version of Htt (Htt103QP) is favored over the soluble one (Htt25QP) in some mutants with reduced endocytosis, we performed a competition assay where equal cell numbers of two isogenic strains expressing *HTT25QP* or *HTT103QP*, were mixed and then grown for five days with dilutions to prevent saturation of the culture (Fig. 7a). Samples were taken every 24 hours and two different techniques were used to quantify competition; a PCR-based method to distinguish the abundance of *HTT103QP* and *HTT25QP* gene copies and Western blots to determine the abundance of the corresponding proteins. Both gene copy number and protein level analysis showed clearly that in wild type cells the aggregating Htt103QP is under negative selection as cells producing Htt103QP are rapidly outcompeted by cells producing the soluble Htt25QP (Fig. 7b). In contrast, the mutants *myo5Δ*, *rvs161Δ*, and *arc18Δ* showed the opposite pattern, where cells producing Htt103QP were in excess after five days of cultivation (Fig. 7b). The change in ratio between the two strains over the five days is presented in Fig. 7b, where a ratio higher than 1 indicates a positive selection for *HTT103QP* expressing cells and ratios lower than 1 a selection for *HTT25QP* expressing cells. These results indicate that in genetic backgrounds displaying a low activity in actin-dependent endocytosis, there is a positive selection for polyQ expansion in Htt. Un-induced cultures (with raffinose as carbon source) were analyzed in parallel to ensure that the differences observed were due to the production of Htt proteins rather than a difference in the propagation of plasmids. No difference in the *HTT103QP*/*HTT25QP* ratio could be detected over the generations in either wild type or mutant cells in these experiments demonstrating that the selections observed were due to the production of Htt proteins (Fig. 7c).

## Discussion

**Figure 8:**
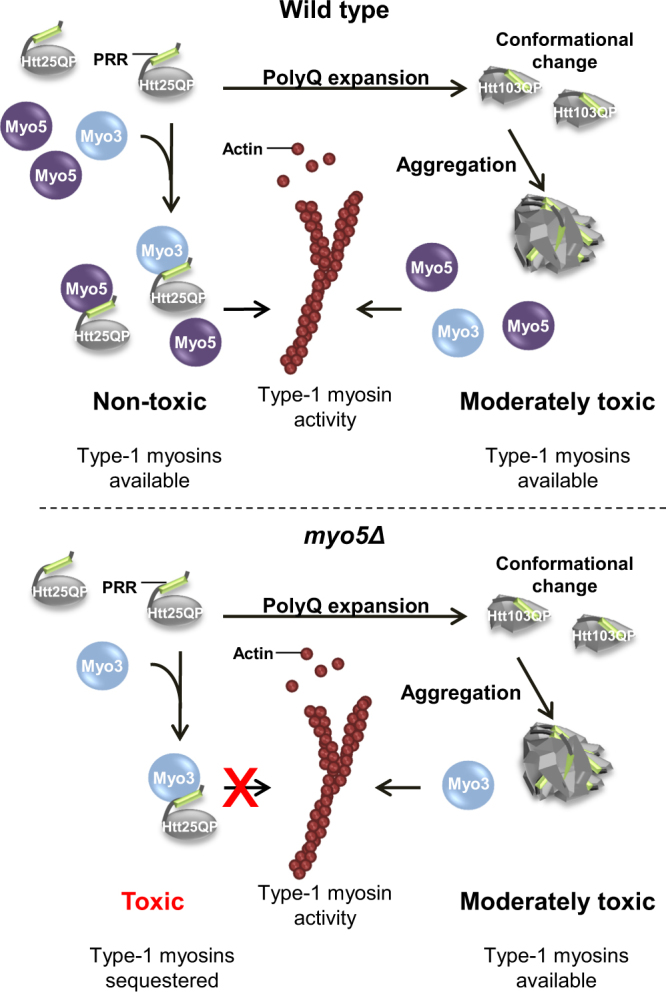
Model for how polyQ expansion may alleviate polyQ toxicity. Soluble Htt25QP can interact with cellular components and prevent them from performing their normal functions, here illustrated by the binding of Htt25QP via its proline-rich region (PRR) to type-1 myosins involved in clathrin-mediated endocytosis. Under normal conditions, in a wild type strain, this interaction and sequestering is buffered by the presence of both type-1 myosins (top). In contrast, when there is a deficiency in type-1 myosins, such as Myo5 (*myo5Δ*), the presence of Htt25QP may titrate out certain key factors, like Myo3, and cause toxicity (bottom). A polyQ expansion above the critical threshold within the Htt protein will result in conformational changes of the protein and lead to protein aggregation, potentially masking the proline-rich region of the expanded Htt protein and provide the cell with free and active type-1 myosins (right). (The moderate toxicity of Htt103QP to cells is caused by another mechanism than the limitation of type-1 myosins).

The SGA analysis demonstrated first, that many more functional groups of genes are required to buffer against inherent toxicity of Htt25QP than Htt25Q and second, that many of those groups of genes are functionally related to actin-dependent, clathrin-mediated endocytosis (Fig. 2). We further linked this toxicity of Htt25QP to reduced functions in endocytosis and actin patch polarity (Fig. 5) and expression of *HTT25QP* increases the sensitivity of yeast to Latrunculin A, suggesting a role for Htt in processes mediated by actin. Several proteins involved in actin cytoskeleton-dependent processes contain SH3 domains, a domain that has been shown to interact with specific proline-rich motifs^17, 18, 27, 28^. Thus, interactions between the proline-rich region of exon-1 and SH3 domains of specific proteins could lead to toxicity. However, Sla1, Bzz1, and Rvs167, which all contain SH3 domains, did not interact with Htt25QP suggesting that its interaction with type-1 myosins is more specific (Fig. 8). That Htt25QP might cause limitations in type-1 myosin activity is supported by Htt25QP binding to both Myo5 and Myo3 (Fig. 4) and that overproduction of Myo3 in cells lacking Myo5 led to a reduction of the Htt25QP-induced toxicity and partially suppressed failures in endocytosis and polarity (Fig. 5). The fact that cells lacking Myo5, but not Myo3, displayed sensitivity towards Htt25QP may be due to Myo3 being the less abundant type-1 myosin^19–22^ and that the residual activity provided by Myo5 in *myo3Δ* cells was sufficient even in the presence of interacting Htt25QP.

The toxicity of soluble Htt25QP, as opposed to the aggregating Htt103QP, in yeast mutants expected to display reduced activity in actin- and clathrin-dependent processes, raises the question of whether polyQ expansions could be under selective pressure under some genetic and/or environmental conditions also in humans. Samples from patients with HD have shown that different somatic tissues suffer from different CAG repeat instabilities within an individual, where the brain regions most affected by neuronal death show the greatest repeat size disparity^29, 30^. In addition, it has been suggested that such differential polyQ expansions in tissues and cells cannot be explained simply by differential cell proliferation allowing for variation by stochastic accumulation of CAG extensions^29^. Whether such expansions in some cases may be under positive selection needs to be investigated further.

## Materials and Methods

### Yeast strains and plasmids

Experiments were performed in BY4741 (*MAT* ***a*** *his3Δ1 leu2Δ met15Δ ura3Δ*) which is referred to as wild type. Deletion mutants have the gene of interest replaced by the KanMX4 cassette (EUROSCARF, Frankfurt, Germany) and strains with TAP-tagged genes were from the yeast TAP-tagged ORF library (GE Healthcare, Dharmacon, Lafayette, CO). A wild type strain with a chromosomally integrated plasmid containing p*GPD* GFP (pPW371) was used for binding of GFP to beads. MYO5 was C-terminally tagged with mRuby2-Leu2 (from pFA6a-link-yomRuby2-Leu2) by transformation using a PCR product with 70 bp of homology to the region of interest. The GFP-tagged *HTT* constructs expressed from the *GAL1* promoter in the 2 µ pYES2 vector were described previously^31, 32^. The control plasmid is pYES2-*GFP* ^33^. The 2 µ MoBY plasmids p5587 (vector control) and p5819-*MYO3* were used in *MYO3* overexpression experiments.

### Growth conditions

Cells were grown at 30 °C in Synthetic drop out media containing 2% raffinose. For induction of the GFP proteins, 1% or 2% galactose was added to the growing culture for 4 hours. For culturing TAP-tagged strains, SD complete media containing 2% glucose was used. For drop tests, exponentially growing cells were cultured in SD-ura or SD-ura-leu containing 2% raffinose and diluted to an optical density of 0.1 or 0.5 followed by dilutions. 5 µl of each dilution was spotted onto appropriate solid media containing 2% galactose (1% galactose and 1% raffinose for strains *rvs161Δ* and *rvs167Δ*) for induction of GFP-tagged proteins. Htt toxicity was quantified using ImageJ by calculating the sum of the pixel values in selected areas (RawIntDen) with background subtractions. The degree of toxicity was plotted as 1 - average RawIntDen without background relative to the control for each strain.

### Synthetic genetic array analysis

The plasmids pYES2-*HTT25QP-GFP* and pYES2-*HTT25Q-GFP* (including the control) were transformed into yeast in Y7092 background to construct the query strains. The SGA screens of *HTT25QP* and *HTT25Q* were performed in duplicate as described before^34, 35^. The screens used the SINGER ROTOR HDA Robot (Singer Instrument Co. Ltd., Somerset, United Kingdom). Functional enrichment analysis was performed using the SGD online analysis software GO Term Finder with the SGA-V2 array as the background list.

### Fluorescence imaging

Imaging was performed using standard fluorescence microscopy with a Zeiss Axio Observer.Z1 inverted microscope (Carl Zeiss, Oberkochen, Germany), equipped with an AxioCam MRm Rev.3 camera (Zeiss) and a Plan-Apochromat 100x/1.40 objective (Zeiss).

### Protein extraction

Harvested cells were washed twice in cold water before extraction of proteins. Cells were lysed either by using NaOH and β-mercaptoethanol or by using a FastPrep (MP Bioscience, United Kingdom). When using a FastPrep, 1 volume of cells was mixed with 1.3 volumes of lysis buffer (10 mM Tris-HCl pH 7.8, 150 mM NaCl, 5 mM MgCl_2_, 10% glycerol, 0.5 mM DTT and protease inhibitor), where after the cells were broken by glass beads (500 µm in diameter) by 6 rounds of beating, at speed 6.5 for 30 second intervals with 2 minute incubations on ice in between. The lysates were diluted in 4 cell volumes of lysis buffer and cleared by several rounds of centrifugation at approx. 20000 *g* at 4 °C. Protein concentrations were determined using the DC Protein assay kit (BioRad, Hercules, CA).

### Immunoblotting

Proteins were separated by SDS or Native PAGE and transferred to either a PVDF (Immobilon FL PVDF 0.45 µm, Millipore, Billerica, MA) or a nitrocellulose (Amersham Protran 0.45 µm NC, GE healthcare, Chicago, IL) membranes using standard methods. Antibodies used were anti-GFP (Roche, Basel, Switzerland) and anti-CBP (Millipore). Signals were visualized using an Odyssey IR scanner (LI-CORE Biosciences, Lincoln, NE).

### Native PAGE

Proteins were extracted using the FastPrep method and a total of 20 µg protein was loaded onto each well of a 3–8% Criterion™ XT Tris-Acetate protein gel (BioRad). Protein complexes were resolved on ice at 100 V for approximately 5 hours.

### Sucrose density gradient fractionation

Proteins were extracted using the FastPrep method and 2 mg protein was loaded onto a 10–40% (w/v) continuous sucrose density gradient and size-fractionated by centrifugation at 85,000 g av for 18 hours at 4 °C. Fractions were mixed with SDS loading buffer, boiled and analyzed on NuPAGE (ThermoFisher Scientific, Waltham, MA) SDS gels.

### Latrunculin A and Benomyl sensitivity tests

Exponentially growing wild type cells containing the *GFP*-tagged constructs were cultured in SD-ura 2% raffinose and approximately 7.5 × 10^5^ cells were plated onto solid media containing 2% galactose. The center of the plate contained a filter disc (6 mm diameter from BD BBL (Fisher Scientific)) soaked in 10 µl of 1 mM Latrunculin A (Enzo Life Science, Farmingdale, NY). Plates were incubated at 30 °C for two to three days and the halo area of no growth measured. For Benomyl tests, exponentially growing cells were diluted to an OD of 0.1 or 0.5 followed by serial dilutions of which 5 µl was spotted onto SD-ura 2% galactose solid media containing 0.15 µg/ml benomyl or DMSO. Plates were incubated at 30 °C for three days.

### Endocytosis assay

Exponentially growing cells were induced for 4 hours in 2% galactose to allow for the production of GFP and Htt-GFP proteins. Cells were washed in 5 ml cold YP, re-suspended in 250 µl cold YP containing 20 µg/ml FM4-64 (N-(3-Triethylammoniumpropyl)-4-(6-(4-(Diethylamino) Phenyl) Hexatrienyl) Pyridinium Dibromide; Life Technologies, Carlsbad, CA), and stained in darkness for 30 minutes in ice/water slurry. After being re-suspended in 5 ml cold YP a 1 ml sample, corresponding to time point zero, was washed one more time in cold YP and stored on ice. The remaining cells were spun down and re-suspended in 4 ml pre-warmed YP containing 2% galactose. Endocytosis was initiated by incubating the cells at 30 °C followed by a 30 min chase. Samples (1 ml) were washed twice in cold YP and stored on ice until analysis. Z-stacks were imaged for visible FM4-64 stained vesicle(s).

### *In vitro* binding assay

Igepal (final concentration 0.2%) was added to protein extracts and after being cleared, the extracts were incubated with 20 µl GFP-trap agarose beads (Chromotek, Planegg, Germany) for 45 minutes at 4 °C on a rotary wheel. Beads with immobilized GFP proteins were washed three times in 1 ml of lysis buffer containing 0.2% Igepal followed by a 1 hour incubation at 4 °C on a rotary wheel together with protein extracts from cells with TAP-tagged proteins. Beads were then washed and proteins bound to the beads eluted with SDS sample buffer, boiled, and resolved on NuPAGE SDS gels.

### RT-PCR

Cells were grown in SD-ura with 2% glucose to an OD of ~1.0 followed by total RNA extraction using a hot acid phenol extraction protocol described in ref. 36. A second RNA isolation was performed using RNeasy mini kit (Qiagen, Hilden, Germany). 1 µg of DNase I treated RNA was used as template for cDNA synthesis, with 3.57 µM random hexamers (Thermo scientific) and reverse transcriptase (Thermo scientific). DNA products of *MYO3* and *CUP1* (control) was amplified by standard real-time PCRs using appropriate primers (available on request), SYBR green (BioRad), and the Bio-Rad Q5 system.

### Actin polarization assay

Cells were fixed in 3.7% formaldehyde for 30 minutes at room temperature, washed in PBS, and stained for F-actin with Rhodamine-Phalloidin (Molecular Probes, Eugene, OR) for 90 minutes at room temperature followed by washing. Mother cells having >6 cortical actin patches were scored as depolarized.

### Competition assay

Cells were first grown as individual cultures to mid log in SD-ura 2% raffinose. Equal cell numbers from each culture were then combined in a new culture and samples for PCR and Western blot analysis were taken. Galactose with a final concentration of 2% was added, after which the cells were allowed to grow at 30 °C for five days. Samples were taken before a daily dilution of the culture to an OD of 0.1. For quantifying the gene ratio of *HTT103QP*/*HTT25QP*, primers were designed to anneal outside the polyQ stretch in the *HTT-GFP* constructs (Htt N17 fw: GGCCTTCGAGTCCCTCAAAA and int GFP rev: CTTGTAGTTGCCGTCGTCCT). PCR was performed with purified total DNA from the mixed culture as template. PCR products were subsequently run on a 1% agarose gel. Protein ratios of Htt103QP/Htt25QP were measured in parallel by making a whole cell extract.

### Statistical analysis

Data in graphs are presented as mean +/− standard deviation or standard error of the mean of triplicates and were analyzed using an unpaired two tailed student’s t-test.

## Electronic supplementary material

Supplementary Information
