# Supplementary material for: Differential effects of soluble and aggregating polyQ proteins on cytotoxicity and type-1 myosin-dependent endocytosis in yeast
Source: Sci Rep. 2017 Sep 12;7:11328. doi: 10.1038/s41598-017-11102-6 (PMC5595923; doi:10.1038/s41598-017-11102-6)
Supplement: Supplementary file 1 — Supplementary Information [file 41598_2017_11102_MOESM1_ESM.pdf]

Supplemental materials for:

Differential effects of soluble and aggregating polyQ proteins on cytotoxicity and type-1 myosin-dependent endocytosis in yeast

Lisa L Berglund[1], Xinxin Hao[1], Beidong Liu[2], Julie Grantham[2], Thomas Nyström*[1]

Genetic interactions with *HTT25QP*

Array ORF	Array Name	Score
YDR159W	SAC3	-0,58005
YLR337C	VRP1	-0,48299
YPL178W	CBC2	-0,466
YBL072C	RPS8A	-0,46156
YHR182W	YHR182W	-0,38334
YNL153C	GIM3	-0,36642
YGR283C	YGR283C	-0,35749
YJR075W	HOC1	-0,35589
YER143W	DDI1	-0,33894
YLR192C	HCR1	-0,32941
YFL023W	BUD27	-0,32689
YMR109W	MYO5	-0,30381
YLR119W	SRN2	-0,30223
YAL013W	DEP1	-0,3
YAL002W	VPS8	-0,29248
YDR245W	MNN10	-0,29234
YMR263W	SAP30	-0,28854
YKR101W	SIR1	-0,27367
YGL136C	MRM2	-0,27199
YHR160C	PEX18	-0,26597
YLR200W	YKE2	-0,26001
YLR089C	ALT1	-0,25758
YDR378C	LSM6	-0,25358
YBR189W	RPS9B	-0,2518
YCR009C	RVS161	-0,24839
YBR200W	BEM1	-0,24825
YCR063W	BUD31	-0,24626
YKR020W	VPS51	-0,24356
YGL202W	ARO8	-0,24255
YBR181C	RPS6B	-0,24151
YLR110C	CCW12	-0,23783
YBL016W	FUS3	-0,23694
YPR043W	RPL43A	-0,23532
YNL097C	PHO23	-0,23127
YOL081W	IRA2	-0,22955
YOL004W	SIN3	-0,22674
YBL007C	SLA1	-0,22618
YDR388W	RVS167	-0,22604
YER116C	SLX8	-0,22482
YHR114W	BZZ1	-0,22448
YLR370C	ARC18	-0,22316
YLR087C	CSF1	-0,21609
YBR290W	BSD2	-0,21455
YBR106W	PHO88	-0,2121
YGR038W	ORM1	-0,21201
YPL069C	BTS1	-0,20076
YGL167C	PMR1	-0,18791
YGR078C	PAC10	-0,18615
YGL168W	HUR1	-0,18532
YBR095C	RXT2	-0,18432
YBR095C	RXT2	-0,18388
YGR027C	RPS25A	-0,18135
YGL127C	SOH1	-0,18038
YDL083C	RPS16B	-0,18033
YCR076C	FUB1	-0,18007
YPL101W	ELP4	-0,17945
YCL032W	STE50	-0,17834
YGL219C	MDM34	-0,17738
YLR081W	GAL2	-0,17525
YGR089W	NNF2	-0,17275
YDR005C	MAF1	-0,1712
YJL051W	IRC8	-0,17022
YGL020C	GET1	-0,16963
YKL007W	CAP1	-0,16928
YOR216C	RUD3	-0,16808
YDL081C	RPP1A	-0,16803
YBR139W	YBR139W	-0,1667
YFR030W	MET10	-0,16618
YML063W	RPS1B	-0,16359
YER095W	RAD51	-0,16321
YPL239W	YAR1	-0,16196
YEL031W	SPF1	-0,16034
YMR116C	ASC1	-0,15822
YIR033W	MGA2	-0,15769
YBR122C	MRPL36	-0,15686
YDR101C	ARX1	-0,1568
YPR045C	THP3	-0,15606
YBR057C	MUM2	-0,15598
YJR049C	UTR1	-0,155
YLR048W	RPS0B	-0,15481
YPL174C	NIP100	-0,15432
YGL163C	RAD54	-0,15403
YIL034C	CAP2	-0,15349
YPL157W	TGS1	-0,15234
YIR003W	AIM21	-0,1522
YER083C	GET2	-0,15125

Genetic interactions with *HTT25Q*

Array ORF	Array Name	Score
YAL013W	DEP1	-0,31429
YAL026C	DRS2	-0,21575
YAR050W	FLO1	-0,18429
YBL016W	FUS3	-0,25146
YBL036C	YBL036C	-0,15682
YBL072C	RPS8A	-0,53483
YBR005W	RCR1	-0,15015
YBR095C	RXT2	-0,25421
YBR095C	RXT2	-0,19616
YBR122C	MRPL36	-0,16735
YBR139W	YBR139W	-0,21788
YBR168W	PEX32	-0,15582
YBR189W	RPS9B	-0,17751
YBR200W	BEM1	-0,1841
YCL032W	STE50	-0,23408
YCR043C	YCR043C	-0,21216
YCR063W	BUD31	-0,16966
YCR075C	ERS1	-0,1563
YCR076C	FUB1	-0,26186
YDL133C-A	RPL41B	-0,15721
YDR005C	MAF1	-0,23637
YDR018C	YDR018C	-0,17626
YDR057W	YOS9	-0,3288
YDR073W	SNF11	-0,15586
YDR148C	KGD2	-0,17786
YDR159W	SAC3	-0,57946
YDR281C	PHM6	-0,16818
YDR304C	CPR5	-0,25213
YDR316W	OMS1	-0,2172
YDR406W	PDR15	-0,20791
YEL001C	IRC22	-0,17856
YER143W	DDI1	-0,33086
YFL023W	BUD27	-0,28058
YFR012W	DCV1	-0,16717
YGL060W	YBP2	-0,19094
YGL168W	HUR1	-0,16012
YGL176C	YGL176C	-0,31967
YGL219C	MDM34	-0,18099
YGR023W	MTL1	-0,18213
YGR038W	ORM1	-0,25118
YGR052W	FMP48	-0,15452
YGR183C	QCR9	-0,15672
YGR260W	TNA1	-0,18249
YGR283C	YGR283C	-0,33778
YHL031C	GOS1	-0,15226
YHR045W	YHR045W	-0,18914
YHR160C	PEX18	-0,37927
YHR182W	YHR182W	-0,298
YIL162W	SUC2	-0,16135
YIR003W	AIM21	-0,20237
YIR033W	MGA2	-0,16811
YJL066C	MPM1	-0,39602
YJL089W	SIP4	-0,18139
YJL216C	IMA5	-0,20121
YJR075W	HOC1	-0,22417
YJR118C	ILM1	-0,15692
YKL041W	VPS24	-0,24988
YKL077W	YKL077W	-0,18119
YKL079W	SMY1	-0,16053
YKL137W	CMC1	-0,1681
YKL157W	APE2	-0,23341
YKR001C	VPS1	-0,21581
YKR020W	VPS51	-0,19824
YKR035W	DID2	-0,16827
YKR049C	FMP46	-0,16215
YKR072C	SIS2	-0,16167
YLL028W	TPO1	-0,23441
YLR039C	RIC1	-0,18468
YLR048W	RPS0B	-0,21069
YLR081W	GAL2	-0,20051
YLR082C	SRL2	-0,16193
YLR087C	CSF1	-0,25167
YLR089C	ALT1	-0,32916
YLR190W	MMR1	-0,29741
YLR192C	HCR1	-0,27235
YLR320W	MMS22	-0,15539
YLR342W	FKS1	-0,17453
YML062C	MFT1	-0,19875
YML063W	RPS1B	-0,1864
YMR263W	SAP30	-0,38159
YNL097C	PHO23	-0,24381
YNL116W	DMA2	-0,34414
YNL117W	MLS1	-0,18078
YOR001W	RRP6	-0,17194
YOR084W	LPX1	-0,17286
YOR312C	RPL20B	-0,1518
YPR024W	YME1	-0,15598
YPR060C	ARO7	-0,15928
YPR145W	ASN1	-0,15328

Table S1. Complete list of genes showing a negative genetic interaction with *HTT25QP* and *HTT25Q*.

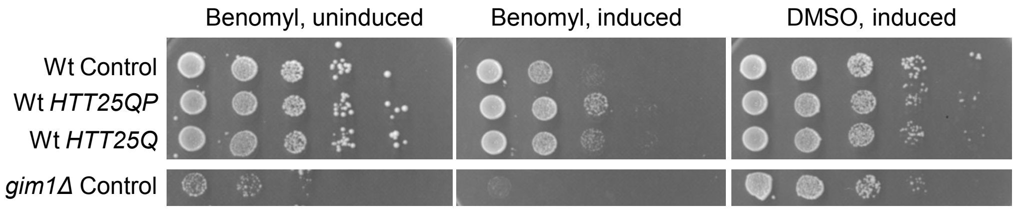

Figure S1. Wild type cells expressing *HTT25QP* are not sensitive to the microtubule depolymerizing drug benomyl. 10x serial dilutions of wild type and *gim1Δ* cells harboring indicated constructs. Cells were grown on selective solid media containing 0.15 µg/ml benomyl or DMSO (control) with 2% glucose (uninduced), to confirm equal cell densities, or 2% galactose (induced), for expression of GFP proteins. The *gim1Δ* cells are sensitive to benomyl and verifies the activity of the drug.

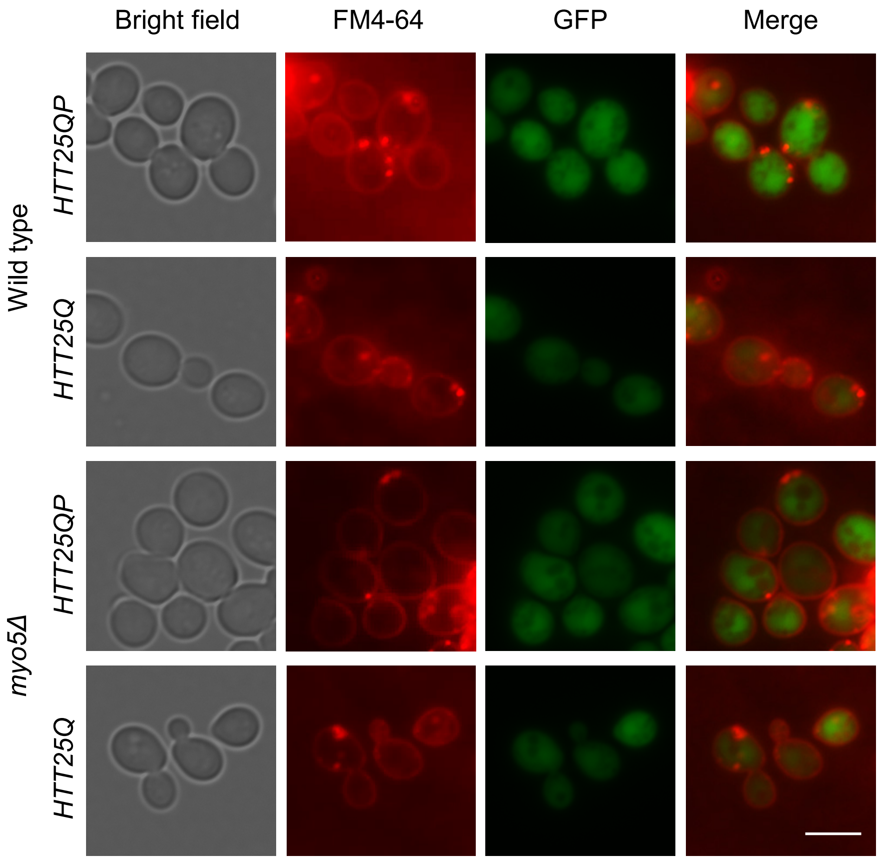

Figure S2. The plasma membrane of wild type and *myo5Δ* cells expressing Htt25QP and Htt25Q can be labelled with FM4-64. Micrographs of indicated cells expressing Htt proteins labelled with FM4-64 and imaged at time point 0 min. Scale bar = 5 µm.

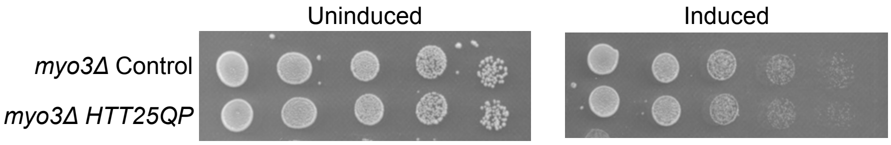

Figure S3. Htt25QP is not toxic in a strain lacking *MYO3*. 5x serial dilutions of *myo3Δ* cells harboring indicated constructs. Cells were grown on selective solid media with 2% glucose, to confirm equal cell densities, or 2% galactose, for expression of GFP proteins.

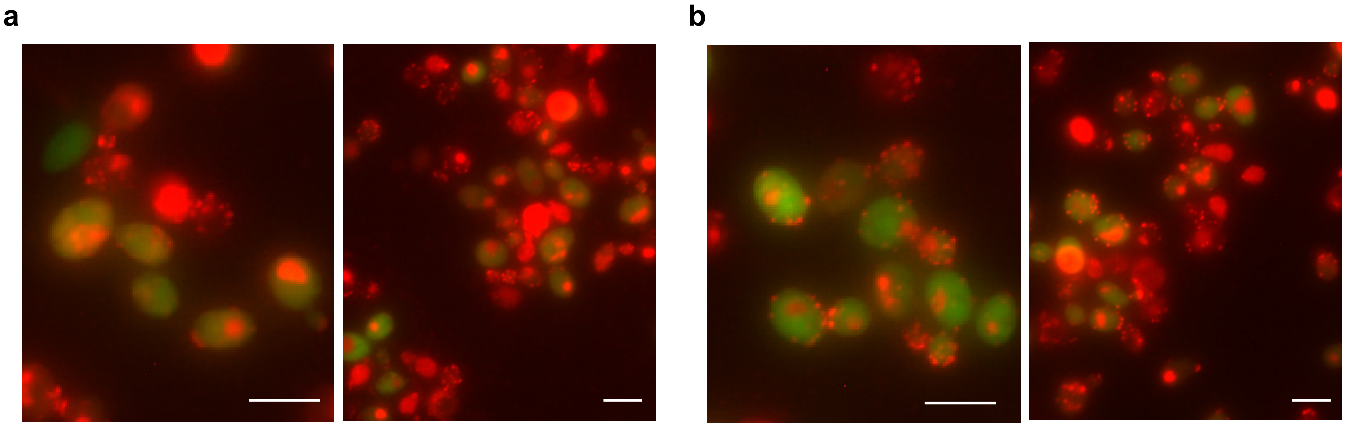

Figure S4. **Cells expressing *HTT25QP* have less Myo5-mRuby localized in patches at the plasma membrane compared to cells expressing *HTT25Q* .** Merged micrographs of wild type cells with Myo5-mRuby (red) expressing **a)** *HTT25QP* (green) or **b)** *HTT25Q* (green). Images are maximum projections of six frames. Scale bar = 5 µm.

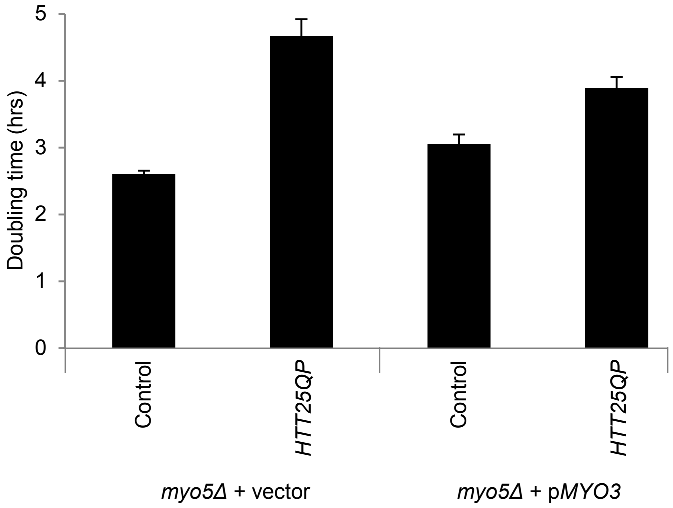

Figure S5. Overexpression of *MYO3* partly suppresses the increased doubling time of *myo5Δ* cells expressing *HTT25QP*. Indicated strains were grown in appropriate liqiuid media with galactose as carbon source and the doubling time was calculated during the exponential growth phase. Values are average from three individual experiments with error bars corresponding to the standard deviation.

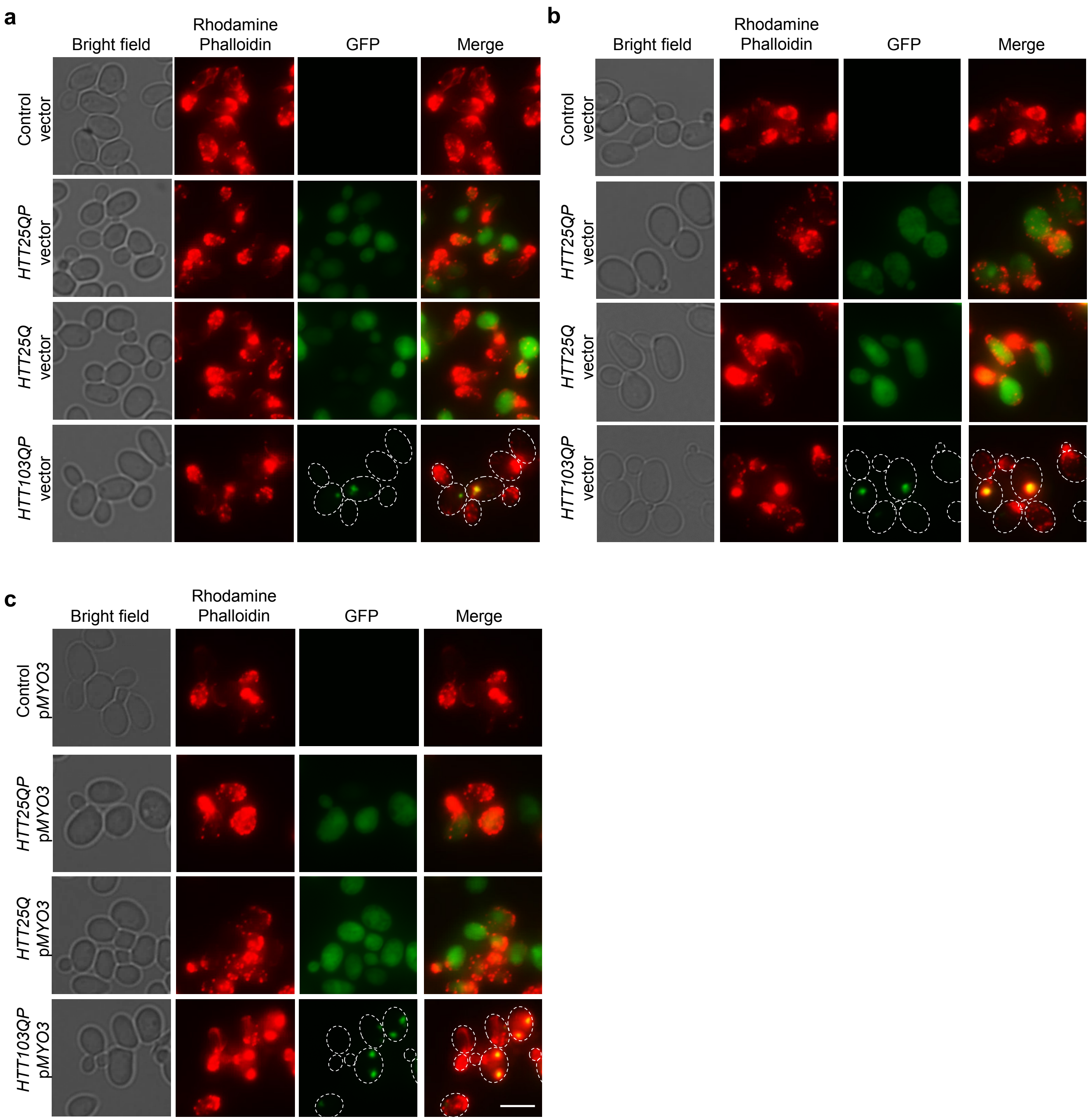

Figure S6. Expression of *HTT25QP* in cells lacking Myo5 results in depolarized actin cytoskeleton. Fluoresence micrographs of **a)** wild type cells with empty vector, **b)** *myo5Δ* cells with empty vector, and **c)** *myo5Δ* cells overexpressing *MYO3,* expressing indicated constructs and stained for F-actin structures using Rhodamine Phalloidin. Scale bar = 5 µm.

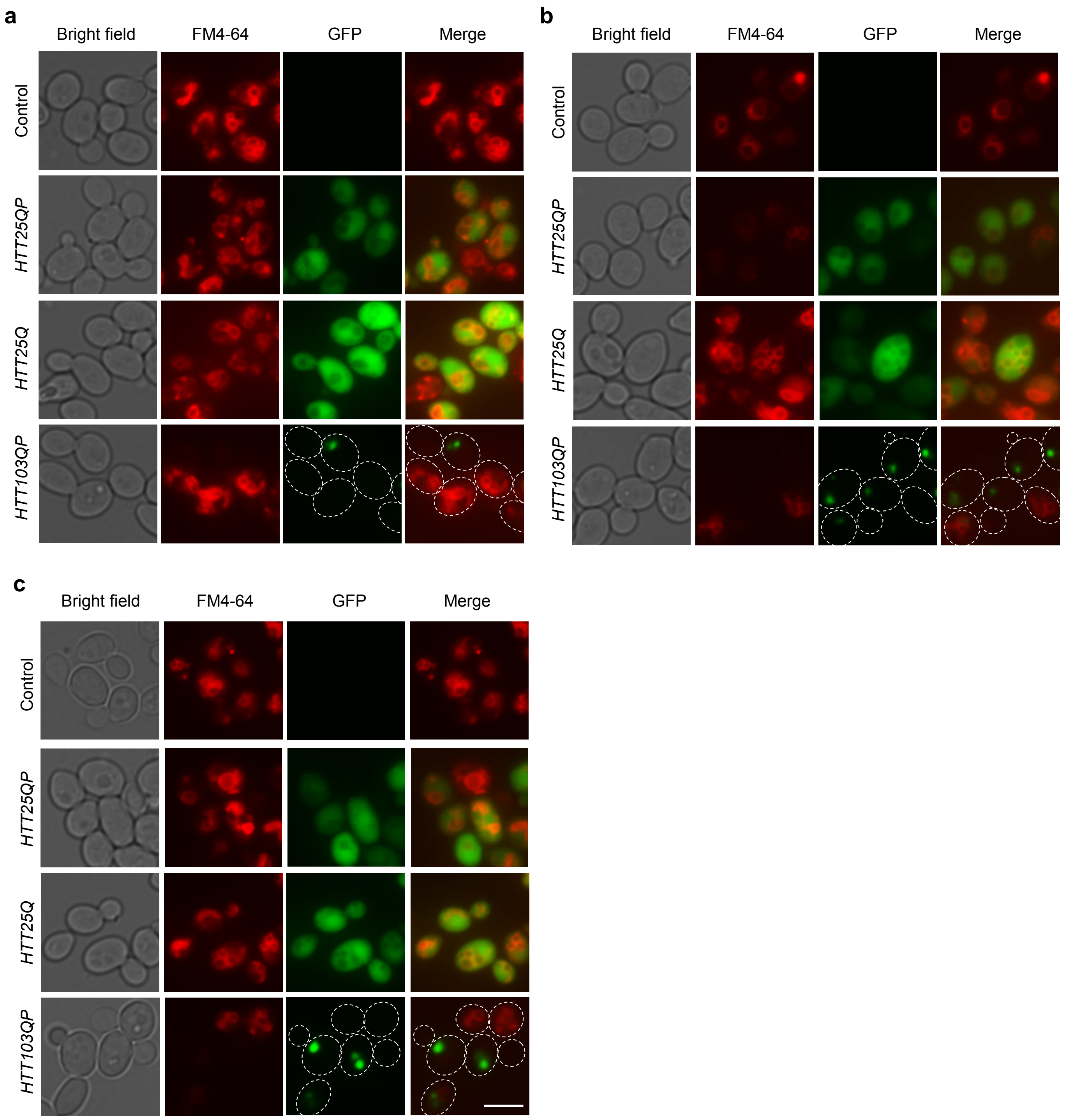

Figure S7. Expression of *HTT25QP* in *myo5Δ* cells and expression of *HTT103QP* in wild type and *myo5Δ* cells results in a decresed endocytic uptake. Fluoresence micrographs of **a)** wild type cells with empty vector, **b)** *myo5Δ* cells with empty vector, and **c)** *myo5Δ* cells overexpressing *MYO3,* expressing indicated constructs and their capability of endocytic uptake. Scale bar = 5 µm.

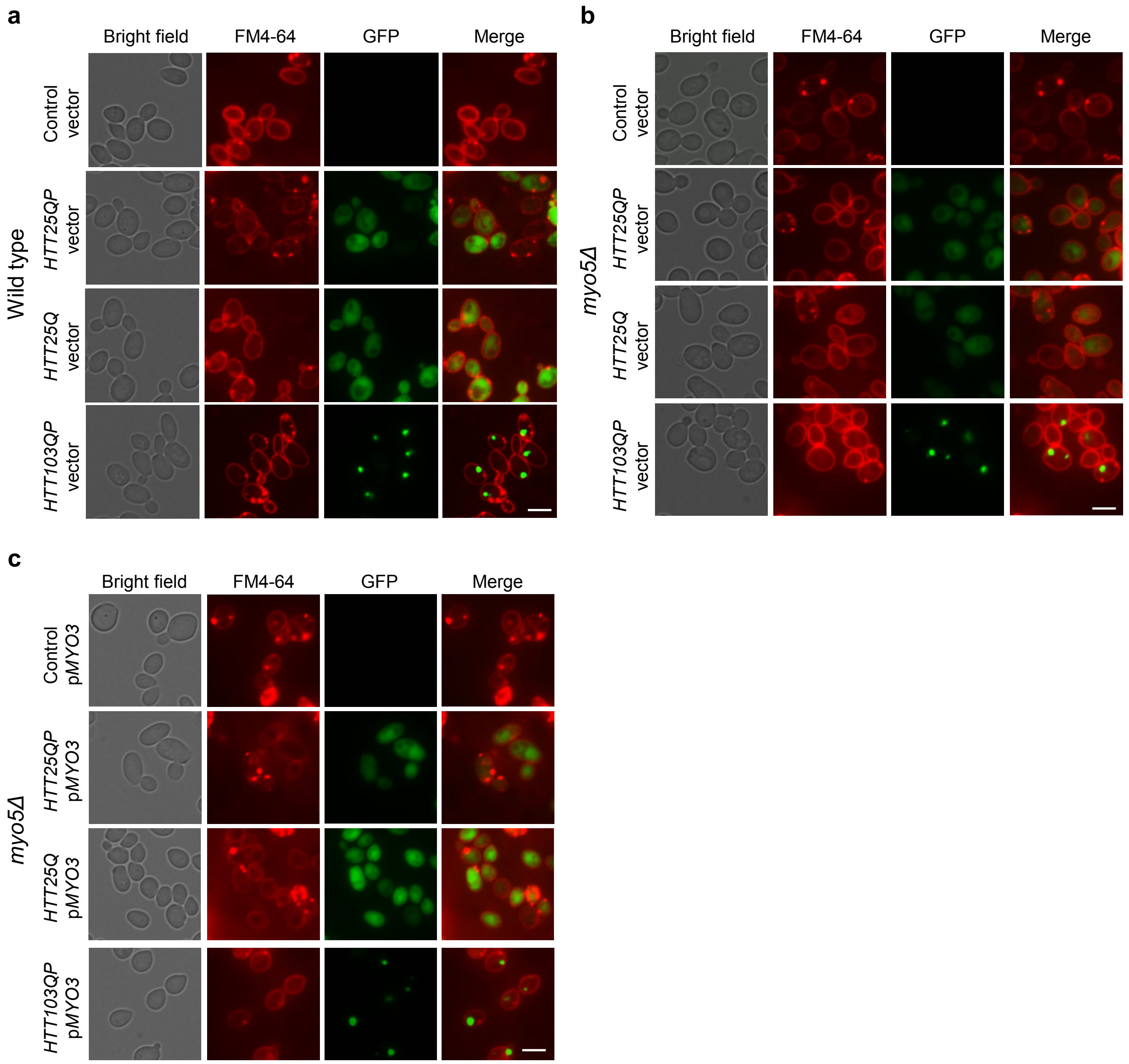

Figure S8. **Expression of the *HTT* constructs used in this study does not affect the FM4-64 labelling of the plasma membrane in any of the strains analyzed.** Fluorescence micrographs of **a)** wild type cells with empty vector, **b)** *myo5Δ* cells with empty vector, and **c)** *myo5Δ* cells overexpressing *MYO3,* expressing indicated constructs labelled with FM4-64 and imaged at time point 0 min. Scale bar = 5 µm.

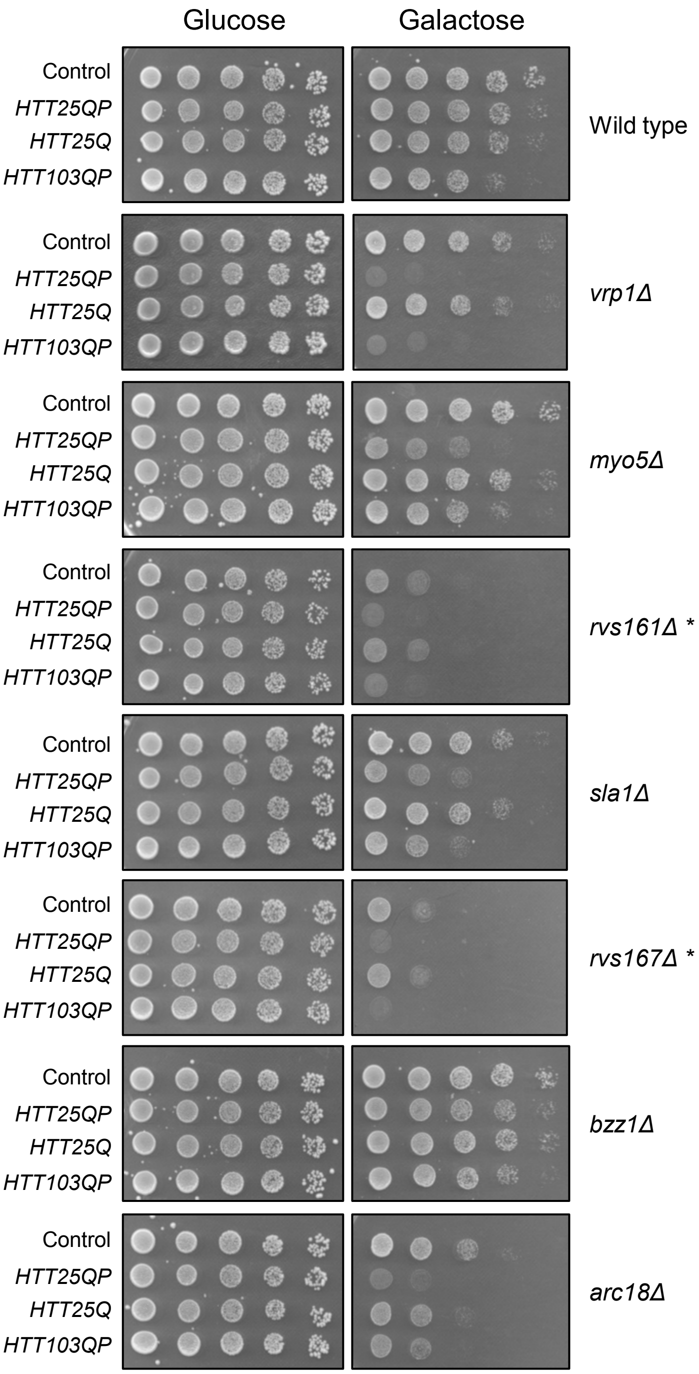

Figure S9. Drop tests showing differential toxicity of Htt25QP and Htt103QP in cells with reduced activities in actin-dependent endocytosis. 5x serial dilutions of strains harboring indicated constructs. Cells were grown on selective solid media containing 2% glucose for confirming equal cell densities (left) or 2% galactose for expression of GFP proteins (right). Representative images are from three individual experiments. * = 1% raffinose and 1% galactose used for expression of GFP proteins

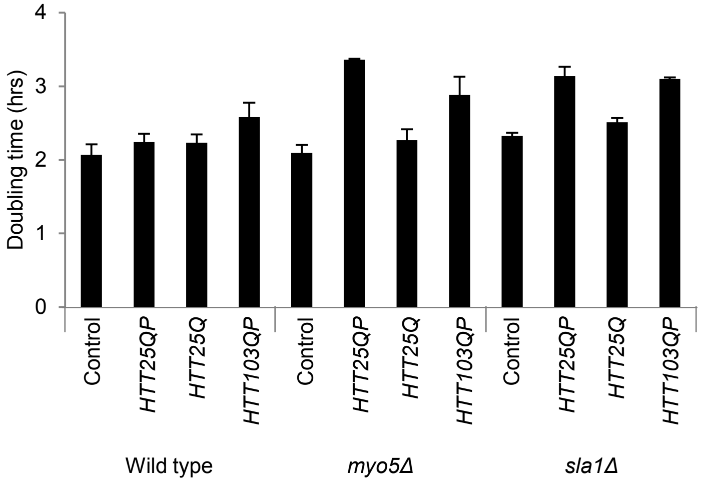

Figure S10. Cells expressing *HTT25QP* and lacking either *MYO5* or *SLA1* display an increased generation time. Indicated strains were grown in appropriate liqiuid media with galactose as carbon source and the doubling time was calculated during the exponential growth phase. Values are average from three individual experiments with error bars corresponding to the standard deviation.

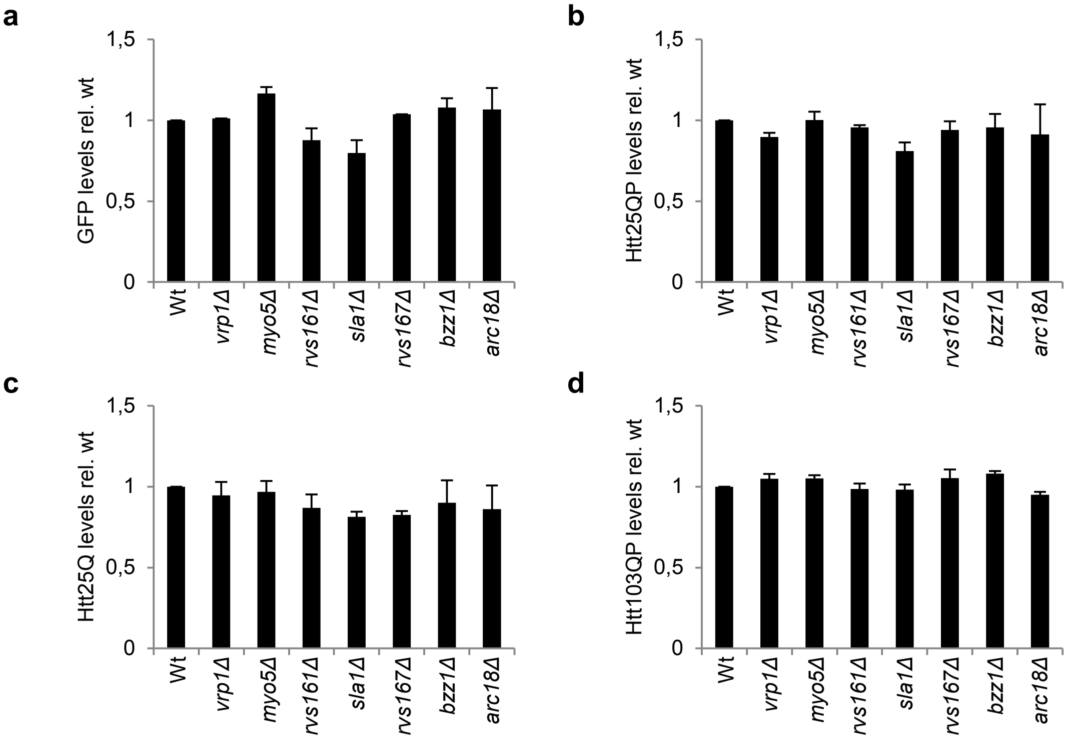

Figure S11. Each of the Htt proteins are expressed at similar levels between the different strains analyzed. Total protein levels of indicated strains and constructs were analyzed with Coomassie brilliant blue and Htt protein levels were visualized by western blotting and probing with anti-GFP antibody. Protein levels of GFP **a)**, Htt25QP **b)**, Htt25Q **c)**, and Htt103QP **d)** normalized to total protein levels. Error bars corresponds to standard deviation.

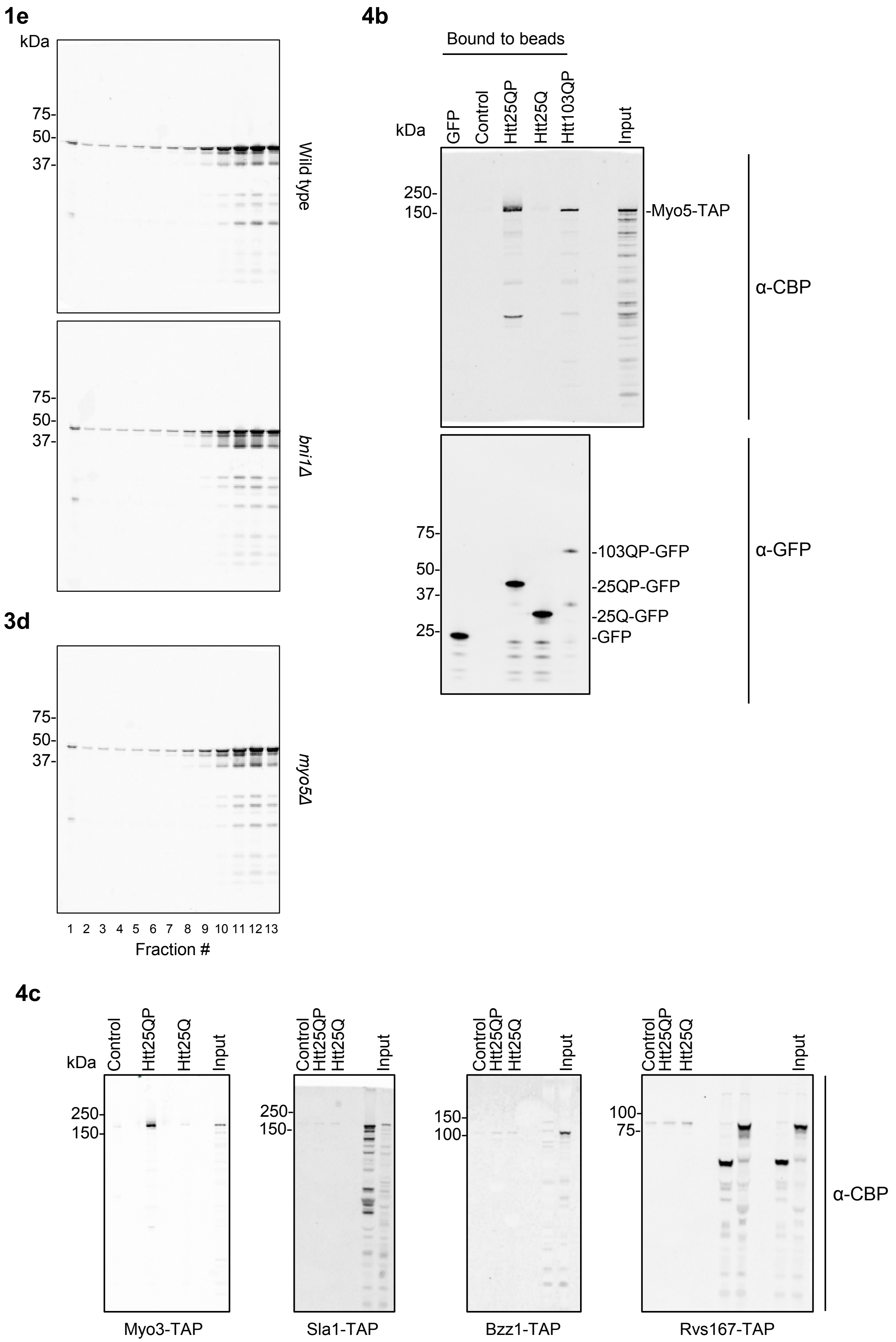

Figure S12. Full-length blots in Fig. 1, 3, and 4

7b, c

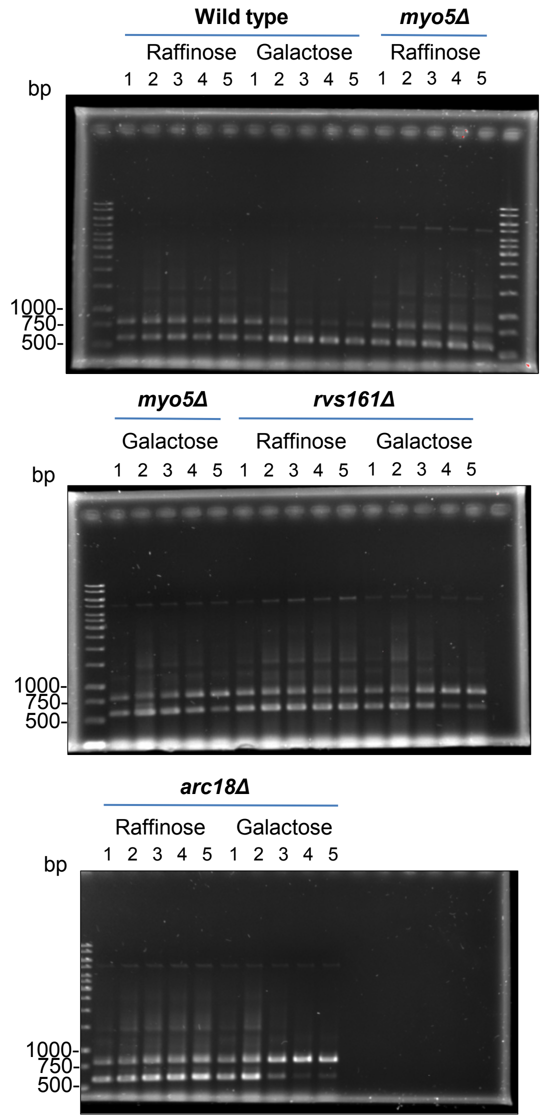

7b

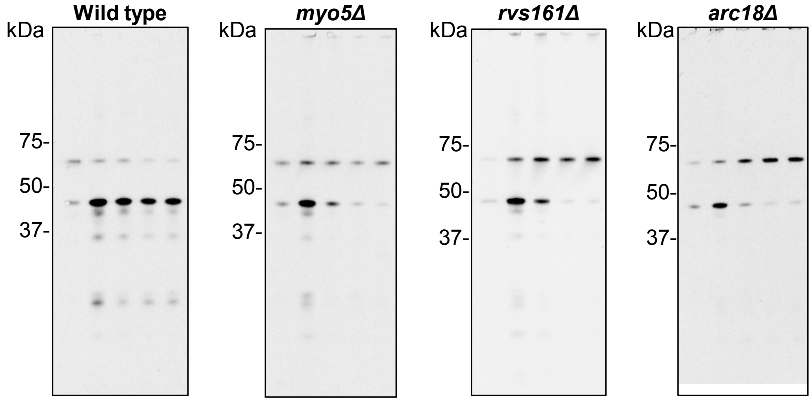

Figure S13. Full-length gels and blots in Fig. 7
